# Protective effect of bixin on carbon tetrachloride-induced hepatotoxicity in rats

**DOI:** 10.1186/0717-6287-47-49

**Published:** 2014-09-29

**Authors:** Priscila R Moreira, Marcos A Maioli, Hyllana CD Medeiros, Marieli Guelfi, Flávia TV Pereira, Fábio E Mingatto

**Affiliations:** Laboratório de Bioquímica Metabólica e Toxicológica (LaBMeT), UNESP – Univ Estadual Paulista, Campus de Dracena, Dracena, SP 17900-000 Brazil; Laboratório de Morfologia da Placenta e Embrião (L@MPE), UNESP – Univ Estadual Paulista, Campus de Dracena, Dracena, SP 17900-000 Brazil

**Keywords:** Bixin, Carbon tetrachloride, Hepatotoxicity, Oxidative stress, Protective activity

## Abstract

**Background:**

The liver is an important organ for its ability to transform xenobiotics, making the liver tissue a prime target for toxic substances. The carotenoid bixin present in annatto is an antioxidant that can protect cells and tissues against the deleterious effects of free radicals. In this study, we evaluated the protective effect of bixin on liver damage induced by carbon tetrachloride (CCl_4_) in rats.

**Results:**

The animals were divided into four groups with six rats in each group. CCl_4_ (0.125 mL kg^-1^ body wt.) was injected intraperitoneally, and bixin (5.0 mg kg^-1^ body wt.) was given by gavage 7 days before the CCl_4_ injection. Bixin prevented the liver damage caused by CCl_4_, as noted by the significant decrease in serum aminotransferases release. Bixin protected the liver against the oxidizing effects of CCl_4_ by preventing a decrease in glutathione reductase activity and the levels of reduced glutathione and NADPH. The peroxidation of membrane lipids and histopathological damage of the liver was significantly prevented by bixin treatment.

**Conclusion:**

Therefore, we can conclude that the protective effect of bixin against hepatotoxicity induced by CCl_4_ is related to the antioxidant activity of the compound.

## Background

The liver is the main organ involved in the biotransformation of exogenous substances (xenobiotics) and has the ability to convert hydrophobic compounds into water-soluble ones that are more easily eliminated by the body [1]. The activity of the liver in the metabolism of xenobiotics is mediated primarily by cytochrome P450 (CYP 450). Although biotransformation reactions are associated with the detoxification process, in some cases, the metabolism of xenobiotics is detrimental to cells due to the production of highly reactive metabolites that are more toxic than the parent compound, such as electrophiles, radicals and reactive oxygen species (ROS), that can react directly with cellular macromolecules or initiate chain reactions [2].

Carbon tetrachloride (CCl_4_) is a small, lipophilic molecule that spreads easily in the lipid compartments of the body and is metabolized in the liver. Its mechanism of toxicity requires a CYP 450-mediated bioactivation step that produces free radicals, such as trichloromethyl (CCl_3_^.^) [3], and induces the peroxidation of lipids. These lipids then damage the membranes of organelles and liver cells, causing the swelling and necrosis of hepatocytes and resulting in the release of cytosolic enzymes such as alanine aminotransferase (ALT) and aspartate aminotransferase (AST) into the circulating blood (Singh *et al.*, 1998; [4, 5]). Due to these properties, CCl_4_ is a chemical that is widely used to induce liver damage in experimental studies [6–10].

The species *Bixa orellana* L. belongs to the family Bixaceae and is popularly known as annatto. The main use of annatto is as a dye. Among the natural colors, annatto is most used by the food industry, especially in the preparation of butter, cheeses, bakery products, oils, ice cream, cereals and meats [11, 12]. In addition to its use in coloring, annatto is also used in folk medicine for the treatment of coronary diseases, disorders of the stomach and intestine, respiratory disorders, burns, and as an aphrodisiac. Annatto leaves are used to fight kidney disease and fever [13, 14], and the tincture prepared from the leaves, immature fruit and flower organs have been shown to present antimicrobial activity [15]. Recently, [16] demonstrated that the aqueous extract of the seeds of *Bixa orellana* was capable of reversing the hypertriglyceridemia induced by Triton, fructose and ethanol, demonstrating a hypolipidemic effect.

The main product of annatto is the seed, from which bixin, a dye of group of carotenoids of great interest in national and international markets, is extracted. Bixin is one of the most effective suppressors of biological molecular oxygen and can protect cells and tissues against the harmful effects of free radicals; additionally, bixin is also an effective inhibitor of lipid peroxidation [17–19]. In the present study, we aimed to investigate the potential effects of bixin in reducing damage and oxidative stress and in improving histopathological abnormalities in the liver of rats treated with CCl_4_ in order to determine the potential of this compound for the treatment or prevention of liver disease.

## Results

### Analysis of alanine aminotransferase (ALT) and aspartate aminotransferase (AST) enzyme activities

**Figure 1:**
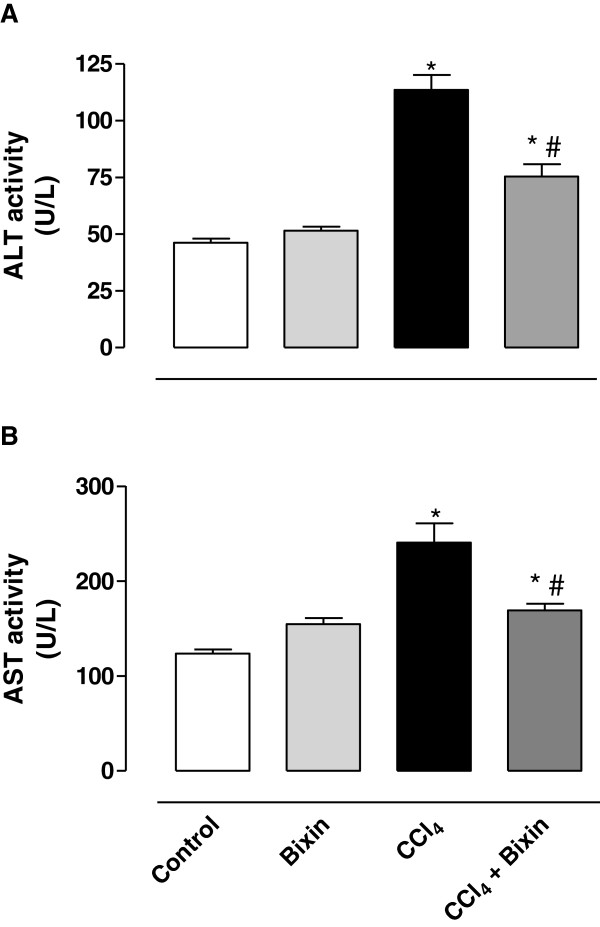
**The effects of bixin on CCl**_**4**_-**induced hepatotoxicity evaluated by ALT (A) and AST (B) activities in the serum.** The results represent the mean ± SEM of six animals per group. * Significantly different from the control group (P < 0.05). ^#^ Significantly different from the CCl_4_-only group (P < 0.05).

Figure 1 shows the effect of bixin on ALT (A) and AST (B) activities in serum. Hepatotoxicity was verified by a significant increase in ALT and AST activities in the CCl_4_–treated group compared with the control group. Pretreatment with bixin significantly prevented the release of these enzymes compared with the CCl_4_–only treated group in serum. The prevention was only partial since the activities of the enzymes from rats treated with CCl_4_ and bixin are significantly different from control. Bixin alone did not have any effect on enzyme activity.

### Lipid peroxidation

**Figure 2:**
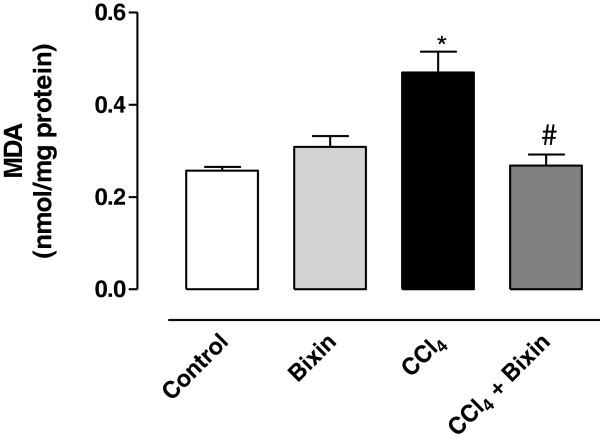
**The effects of bixin on CCl**_**4**_ **-induced malondialdehyde (MDA) generation in rat liver homogenate.** The results represent the mean ± SEM of six animals per group. * Significantly different from the control group (P < 0.05). ^#^ Significantly different from the CCl_4_-only group (P < 0.05).

A significant increase in the level of MDA, an end product of lipid peroxidation, was observed in the liver of CCl_4_–treated rats when compared with the control group (Figure 2). Pretreatment with bixin significantly prevented the MDA production in the liver when compared with that of rats administered CCl_4_. Bixin alone did not have any effect on this parameter.

### Determination of GSH and NADPH levels on liver homogenate

**Figure 3:**
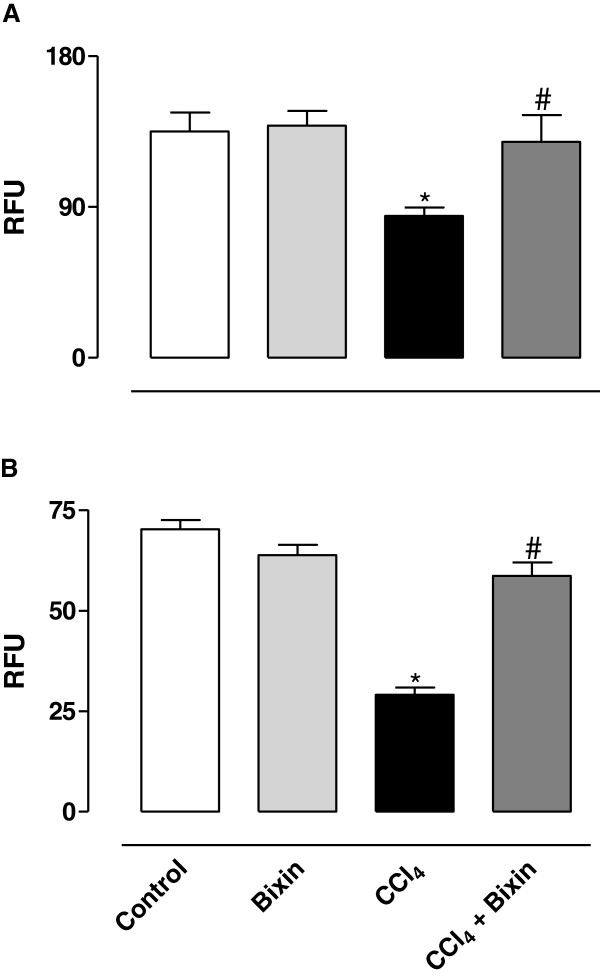
**The effects of bixin on CCl**_**4**_ **-induced GSH (A) and NADPH (B) oxidation in rat liver homogenate.** The results represent the mean ± SEM of six animals per group. * Significantly different from the control group (P < 0.05). ^#^ Significantly different from the CCl_4_-only group (P < 0.05). RFU: Relative Fluorescence Unit.

Treatment of rats with CCl_4_ significantly reduced the levels of GSH and NADPH in the liver when compared with the control group (Figures 3A and B, respectively). The pretreatment of animals with bixin significantly prevented these changes, maintaining levels of the compounds to the normal range. Bixin alone did not have any effect on these parameters.

### Analysis of glutathione reductase activity

**Figure 4:**
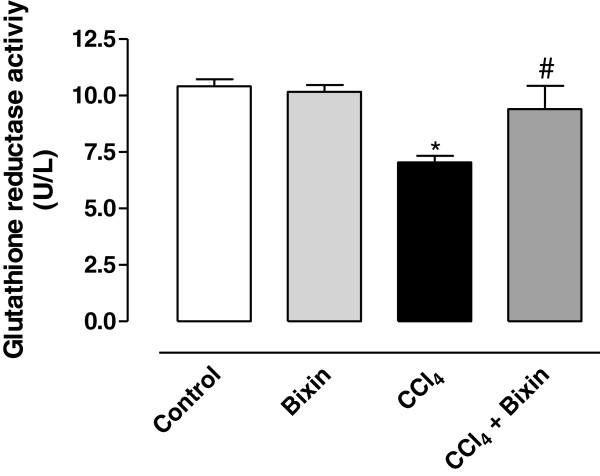
**The effects of bixin on CCl**_**4**_ **-induced reduction of GR activity in rat liver homogenate.** The results represent the mean ± SEM of six animals per group. * Significantly different from the control group (P < 0.05). ^#^ Significantly different from the CCl_4_-only group (P < 0.05).

Analysis of glutathione reductase (GR) activity showed a significant reduction in activity in the CCl_4_–treated group when compared to the control group (Figure 4). Pretreatment of animals with bixin prevented the change in GR activity, showing a protection against the effects of CCl_4_. Bixin alone did not have any effect on enzyme activity.

### Histopathological analysis

**Figure 5:**
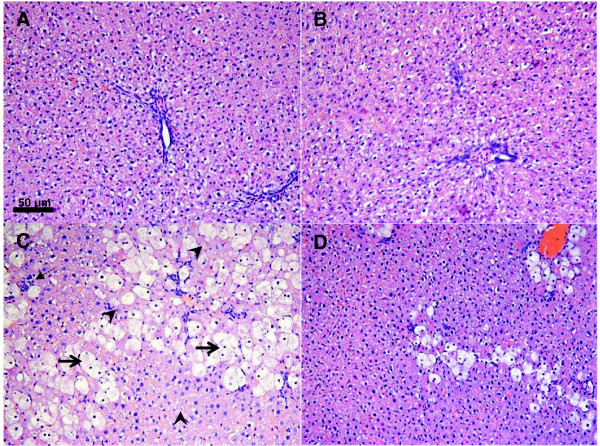
**The effects of bixin on CC1**_**4**_ **-induced liver damage in rat. (A)** liver of control group showing intact liver structures; **(B)** liver of bixin group, showing normal structure; **(C)** liver of CCl_4_ group, showing hepatocytes necrosis (arrow head), hydropic degeneration (arrow) and infiltration of inflammatory cells (dashed arrow); and **(D)** liver of CCl_4_ + bixin showing prevented damage. H&E, original magnification 200 × .

Histopathological studies of the liver of control and bixin-only treated animals showed normal histology (Figures 5A and B, respectively). In animals treated with CCl_4_, inflammation, necrosis and hydropic degeneration of hepatic cells was observed (Figure 5C). The group that was pretreated with bixin showed that severe hepatic lesions induced by CCl_4_ were partially prevented (Figure 5D), which were in agreement with the results of the serum aminotransferases activities and lipid peroxidation.

## Discussion and conclusion

The liver plays an important role in metabolism and biotransformation of xenobiotics. Due to its position between the digestive tract and circulatory system, it receives large amounts of nutrients and xenobiotics absorbed through the digestive tract and the portal vein becoming the target organ of several classes of toxins and toxicants, natural or synthetic [1].

Bixin, a carotenoid with one carboxylic acid and one methyl ester group, is the major pigment found in annatto and corresponds to approximately 80% of the carotenoids present in the plant seed [20, 21]. This compound has been demonstrated to protect cells and tissues against the deleterious effects of ROS and free radicals and also exhibits a cholesterol lowering effect [17, 18, 22–24]. In a previous study, [2] evaluated the role of bixin on cisplatin-induced oxidative stress in the kidneys of Wistar rats at doses of 2.5 or 5.0 mg kg^-1^ body wt. by gavage. Pretreatment with the highest dose of bixin resulted in a reduction in the total number of chromosome aberrations, inhibited increases in lipid peroxidation, and inhibited renal glutathione depletion induced by cisplatin. Therefore, in our study we decide to evaluate the potential hepatoprotective effects of bixin using this same dose.

CCl_4_ is a hepatotoxin that causes liver damage and has been used in several studies as a hepatotoxic agent [5, 8, 25–28]. The most striking pathological features of CCl_4_-induced hepatotoxicity are hepatic steatosis, cirrhosis and necrosis, which results from the formation of reactive radical intermediates such as trichloromethyl (CCl_3_^.^) formed by CYP 450-mediated biotransformation that induces membrane lipid peroxidation [3, 29, 30].

The serum activities of alanine transaminase (ALT) and aspartate transaminase (AST) are used as an indication of the extent of liver damage due to the release of large quantities of these enzymes into the bloodstream [31]. AST is distributed in body tissues, including muscle and the heart. In the liver, AST is mainly present in the mitochondria of hepatocytes while the ALT is found outside of the mitochondria. CCl_4_ induces the peroxidation of lipids that damage the membranes of liver cells and organelles and results in the release of ALT and AST into the circulating blood [5]. Accordingly, our results demonstrated that treatment with CCl_4_ promoted a significant increase in ALT and AST serum activities. Pretreatment with bixin protected the liver from damage by CCl_4_, as there was a significant decrease in the release of enzymes.

As mentioned above, the hepatotoxic effects of the metabolism of CCl_4_ are mainly due to its active metabolite, the trichloromethyl radical, which in the presence of oxygen, is transformed into trichloromethyl peroxyl radical (CCl_3_OO^.^). These free radicals bind covalently to macromolecules and induce peroxidative degradation of membrane lipids that are rich in polyunsaturated fatty acids [23]. This leads to the formation of lipid peroxides that give rise to products such as malondialdehyde (MDA) that cause damage to the membranes [32, 33]. The increased MDA in the liver of CCl_4_-treated rats suggests that the natural antioxidant defense mechanism to scavenge excessive free radicals has been compromised. However, pretreatment with bixin significantly prevented the formation of MDA, indicating hepatoprotection by impairing initiation and propagation of the peroxidative process.

To protect itself from oxidation damage, the cell has a defense system that includes reduced glutathione (GSH), nicotinamide adenine dinucleotide phosphate in the reduced form (NADPH) and enzymes such as superoxide dismutase (SOD), catalase, glutathione peroxidase (GPx) and glutathione reductase (GR). The imbalance between the formation and removal of free radicals in the body, due to the reduction of endogenous antioxidants or increased generation of oxidizing species, generates a pro-oxidant condition known as oxidative stress, which favors the occurrence of oxidative lesions in macromolecules and cellular structures, and possibly result in cell death [34]. Under conditions of oxidative stress, some of the endogenous protective factors decrease. Accordingly, treatment of animals with CCl_4_ caused a significant decrease in the levels of GSH and NADPH, and in the activity of the GR. However, we observed a protective effect of bixin on oxidative stress caused by CCl_4_ because the pretreatment of animals with the compound prevented the oxidation of GSH and NADPH and the reduction of the activity of GR, indicating an indirect antioxidant property of the bixin, probably by reacting with the free radicals arising from CCl_4_ as demonstrated by [35].

The histological observations in the liver samples strongly support the protective effect of bixin. CCl_4_ caused various histological changes to the liver, including cell necrosis, inflammation, and hydropic degeneration of hepatic cells. These alterations were significantly attenuated by bixin in livers, resulting in only minor hepatocellular necrosis and inflammatory cell infiltration. In conclusion, our results indicate that pretreatment with bixin attenuated liver injury produced by carbon tetrachloride while significantly reducing the alterations caused by ALT and AST activities, lipid peroxidation, and histopathological parameters. Additionally, bixin protected against reduction of GSH and NADPH levels and GR activity. These effects may be related to the antioxidant activity of bixin.

## Methods

### Chemicals

Annatto powder (containing 28% bixin, determined spectrophotometrically) was kindly supplied by Christian Hansen Indústria e Comércio Ltda (Valinhos, SP, Brazil). The amount of annatto supplied to the animals was adjusted to contain the desired dose of bixin. All other reagents were of the highest commercially available grade.

### Animals

Male Wistar rats weighing approximately 200 g were used in this study. The animals were obtained from the Central Bioterium of UNESP – Univ Estadual Paulista, Campus de Botucatu, SP, Brazil, and were maintained with a maximum of 4 rats per cage under standard laboratory conditions with water and food provided *ad libitum*. The experimental protocols were approved by the Ethical Committee for the Use of Laboratory Animals of the UNESP – Univ Estadual Paulista, Campus de Dracena, SP, Brazil.

### Treatment

The animals were divided into four groups, each with 6 animals. Group 1 was the control group, and received canola oil by gavage for 7 days and mineral oil intraperitoneally on the last day. Group 2 was treated with bixin suspended in canola oil (5.0 mg/kg body weight) by gavage for 7 days before mineral oil application. Group 3 received canola oil by gavage for 7 days and CCl_4_ dissolved in mineral oil (0.125 mL/kg body weight), administered intraperitoneally, on the last day. Group 4 received bixin suspended in canola oil (5.0 mg/kg body weight) by gavage for 7 days and carbon tetrachloride, administered intraperitoneally, on the last day. Twenty-four hours after the administration of the vehicle or CCl_4_, animals were euthanized by decapitation and biochemical and histopathological analyses were performed. The dose of bixin (5.0 mg/kg body weight) used in this study as pre-treatment, was based on data found in the literature as showing protective effects in the kidney [36].

### Analysis of enzymes indicative of hepatic functions

Blood samples were collected and kept at room temperature for 15 minutes to allow for coagulation. Serum was separated by low-speed centrifugation (3000 g for 15 min), and the activity of the enzymes ALT and AST was measured using commercially available kits (Bioclin, Belo Horizonte, Brazil) according to the manufacturer's protocols.

### Preparation of rat liver homogenate

The liver was removed, sliced into 50 mL of medium (250 mM sucrose, 1 mM EGTA and 10 mM HEPES-KOH, pH 7.2) at 4°C, washed three times with the same medium and homogenized three times for 15 sec at 1 min intervals with a Potter-Elvehjem homogenizer. The protein concentration of the homogenate was determined by the biuret reaction with BSA as a standard [37].

### Membrane lipid peroxidation (LPO) assay

The level of LPO was estimated by malondialdehyde (MDA) generation [38]. The liver homogenate (5 mg of protein) was added to a tube. Following the addition of 0.2 mL of 8.1% SDS, 1.5 mL of 20% acetic acid and 1.5 mL of 0.67% thiobarbituric acid (TBA, aqueous solution), glass-distilled deionized water was added to a final volume of 4 mL. The mixture was incubated for 60 min at 85°C. The MDA-TBA complex was extracted with 5 mL of n-butanol and the absorbance was measured at 535 nm. The MDA concentration was calculated with ϵ = 1.56 × 10^5^ M^-1^ cm^-1^.

### Determination of GSH level

Liver homogenate (1 mg of protein) was added to medium (125 mM sucrose, 65 mM KCl and 10 mM HEPES-KOH, pH 7.4) to a final volume of 1 mL and treated with 0.5 mL of 13% trichloroacetic acid. The mixture was stirred and then centrifuged at 9000 g for 3 min. Aliquots (100 μL) of the supernatant were mixed with 2 mL of 100 mM NaH_2_PO_4_ buffer at pH 8.0 containing 5 mM EGTA. One hundred microliters of an o-phthaldialdehyde solution (1 mg/mL) was added, and the fluorescence was measured 15 min later in a spectrofluorometer (Shimadzu-RFPC 5301, Tokyo, Japan) using 350/420 nm as the excitation/emission wavelength pair [39]. The data are expressed in relative units of fluorescence.

### Determination of NADPH level

Liver homogenate (1.5 mg protein) was added to medium (125 mM sucrose, 65 mM KCl and 10 mM HEPES-KOH, pH 7.4) to a final volume of 1.5 mL and centrifuged at 8000 g for 3 min. The supernatant was collected, and the fluorescence was measured in a spectrofluorometer (Shimadzu-RFPC 5301, Tokyo, Japan) using 366/450 nm as the excitation/emission wavelength pair. The data are expressed in relative units of fluorescence.

### Glutathione reductase activity

One milliliter of 0.1 mM sodium phosphate buffer, pH 7.6, with 0.5 mM EDTA, 10 μL of 10% Triton X-100, liver homogenate (1 mg of protein) and 10 μL of 100 mM GSSG was added to 4 mL quartz cuvettes. After incubating the samples at 30°C for 5 minutes, 10 μL of 10 mM NADPH was added, and the variation in absorbance was determined at a wavelength of 340 nm in a spectrophotometer (Beckman-Coulter model DU-800, Fullerton, CA, USA).

### Histopathological analysis

Liver fragments were fixed in a 10% solution of formaldehyde, dehydrated in graduated ethanol (50-100%), cleared in xylene and embedded in paraffin. The hepatic sections (4–5 μm) were analyzed by light microscopy with a magnification of 200× after staining with hematoxylin and eosin (H&E) using standard techniques.

### Statistical analysis

Significant differences were calculated by one-way analysis of variance (ANOVA) followed by the Tukey test using the GraphPad Prism software, version 4.0 for Windows (GraphPad Software, San Diego, CA, USA). Values of P < 0.05 were considered significant.
